# Xeno-Free 3D Bioprinted Liver Model for Hepatotoxicity Assessment

**DOI:** 10.3390/ijms25031811

**Published:** 2024-02-02

**Authors:** Ahmed S. M. Ali, Johanna Berg, Viola Roehrs, Dongwei Wu, Johannes Hackethal, Albert Braeuning, Lisa Woelken, Cornelia Rauh, Jens Kurreck

**Affiliations:** 1 Department of Applied Biochemistry, Institute of Biotechnology, Technische Universität Berlin, TIB 4/3-2, Gustav-Meyer-Allee 25, 13355 Berlin, Germany; 2 THT Biomaterials, 1030 Vienna, Austria; j.hackethal@tht-biomaterials.com; 3 Department Food Safety, German Federal Institute for Risk Assessment (BfR), 10589 Berlin, Germany; albert.braeuning@bfr.bund.de; 4 Department of Food Biotechnology and Food Process Engineering, Technische Universität Berlin, 14195 Berlin, Germanycornelia.rauh@tu-berlin.de (C.R.)

**Keywords:** 3D bioprinting, xeno-free, liver model, HuH-7 cell line, chemically defined media, FBS-free

## Abstract

Three-dimensional (3D) bioprinting is one of the most promising methodologies that are currently in development for the replacement of animal experiments. Bioprinting and most alternative technologies rely on animal-derived materials, which compromises the intent of animal welfare and results in the generation of chimeric systems of limited value. The current study therefore presents the first bioprinted liver model that is entirely void of animal-derived constituents. Initially, HuH-7 cells underwent adaptation to a chemically defined medium (CDM). The adapted cells exhibited high survival rates (85–92%) after cryopreservation in chemically defined freezing media, comparable to those preserved in standard medium (86–92%). Xeno-free bioink for 3D bioprinting yielded liver models with high relative cell viability (97–101%), akin to a Matrigel-based liver model (83–102%) after 15 days of culture. The established xeno-free model was used for toxicity testing of a marine biotoxin, okadaic acid (OA). In 2D culture, OA toxicity was virtually identical for cells cultured under standard conditions and in CDM. In the xeno-free bioprinted liver model, 3-fold higher concentrations of OA than in the respective monolayer culture were needed to induce cytotoxicity. In conclusion, this study describes for the first time the development of a xeno-free 3D bioprinted liver model and its applicability for research purposes.

## 1. Introduction

The liver is the first line of defense against toxic chemical substances and, as a result, is often the first organ to be affected, making it a critical organ for toxicity testing [1,2]. However, relying on laboratory animals for such tests raises ethical concerns and lacks accurate predictability for human safety [3,4]. The efficacy of translating toxicity testing outcomes from animals to humans exhibits a failure rate exceeding 92% [5]. As a result, there is a growing emphasis on developing alternative, non-animal methodologies (NAMs). Over the years, numerous in vitro liver models have been developed, including 2D cell cultures, microfluidic devices, and 3D organoids [6,7,8,9]. These diverse models serve as valuable tools for studying hepatotoxicity. Nonetheless, each of these models has its specific limitations in accurately reproducing the intricate structure and functionality of the liver.

Another modern technology used to address this need is 3D bioprinting, which involves the precise deposition of living cells and biomaterials in a layer-by-layer manner to create three-dimensional structures that closely imitate the architecture and function of natural liver tissue [10,11,12]. The great promise of 3D bioprinting is its remarkable flexibility and resolution, enabling the creation of human-relevant tissue models, which reduces the necessity for animal testing in biomedical research [13]. It is, however, crucial to acknowledge that prevailing bioprinting techniques frequently rely on the utilization of diverse animal-derived components [14]. In a recent systematic review of published studies using bioprinted liver models, we could not identify a single xeno-free study (unpublished data). The vast majority of studies cultured cells in medium containing FBS, and components of animal origin were used in most bioinks. This, again, raises ethical concerns and leads to the creation of chimeric systems composed of human cells in an animal environment, which limits the biological relevance of the in vitro models. Therefore, both ethical considerations and scientific relevance require more reliable and scientifically well-defined models by transitioning towards xeno-free 3D bioprinting.

One of the primary hurdles in xeno-free bioprinting is the limited availability of cell types adapted to animal serum-free media. Animal sera, particularly fetal bovine serum (FBS, also denoted as fetal calf serum, FCS), have historically been widely used in cell culture due to their unique set of essential growth factors, proteins, vitamins, trace elements, hormones, and other beneficial components [15]. Nonetheless, ethical concerns arise from the realization that the collection of FBS involves cruelty and pain to the fetuses in addition to the risk of pathogen transmission and inconsistency between different FBS batches [16]. In addition, variations between different FBS batches are considered a major problem that limits the reproducibility of cell culture experiments [17].

A potential alternative to FBS is human platelet lysate (HPL), as it promotes the in vitro propagation of many cell types given the advantage of its high content of various growth factors [18,19,20]. However, like FBS, HPL also exhibits batch-to-batch variability owing to its human source [21]. An alternative that can effectively circumvent the issue of batch-to-batch variability is the use of chemically defined media (CDM). By precisely specifying all components in the media, CDM ensure consistent and reproducible culture conditions for cells [22]. Numerous CDM formulations have been documented for mammalian cells and are collected in the FCS-free database (fcs-free.org), and a variety of commercially accessible serum-free media options are currently available. Nevertheless, commercial serum-free media are currently expensive, and CDM are not available for all cell lines and primary cells.

The long-term storage of cells through cryopreservation is another crucial procedure that often involves the use of animal-derived substances like FBS and bovine serum albumin (BSA). Consequently, several formulations of chemically defined freezing media (CDFM) have been documented, wherein dextran, sucrose, Pluronic F68, methylcellulose, polyvinylpyrrolidone (PVP), polyvinyl alcohol, ficoll, or poly(ethylene glycol) replace FBS and BSA [23,24,25,26,27]. Although such formulations are available, there is no guarantee one will work for any given cell type. Therefore, testing different formulations becomes necessary to identify the most suitable one.

Finally, another notable hurdle in xeno-free bioprinting lies in the development of an animal-free bioink. A desirable bioink must create an optimal environment for cell survival, proliferation, and differentiation, closely resembling in vivo conditions to facilitate the formation of functional tissues [28,29]. Consequently, the current formulation of bioinks often relies on the use of xenogeneic materials, e.g., basement membrane extracts (BME, best known under the brand name Matrigel), gelatine, collagen and decellularized extracellular matrix (dECM) [30,31,32,33,34]. To address this, researchers have explored various synthetic polymers as potential alternatives to animal-derived materials [35]. Nevertheless, synthetic polymers have some limitations in replicating the complexity and growth factors provided by natural materials. In pursuit of more promising alternatives, human-derived materials, such as collagen, placenta, and laminin have emerged as viable options for replacing animal-derived materials in the realm of 3D bioprinting [36,37,38]. However, these materials can be difficult to obtain in large quantities, and the composition of the ECM can vary depending on the tissue source [39], so they do not provide a promising alternative.

HuH-7 is one of the most commonly used human cell lines for liver research. It was derived from a hepatocellular carcinoma in 1982 [40]. The HuH-7 cell line enabled important progress towards understanding hepatic viruses. Permissive cells derived from HuH-7 made the in vitro propagation of hepatitis C virus possible for the first time and laid the basis for a deeper understanding of virus biology and the development of antiviral agents [41,42,43]. In addition, the HuH-7 cell line is used in research dealing with Hepatitis E virus [44] and dengue virus [45]. Furthermore, HuH-7 cells are known for their high level of sensitivity to certain drugs and toxins, making them a useful tool for studying liver toxicity in vitro [46].

**Figure 1:**
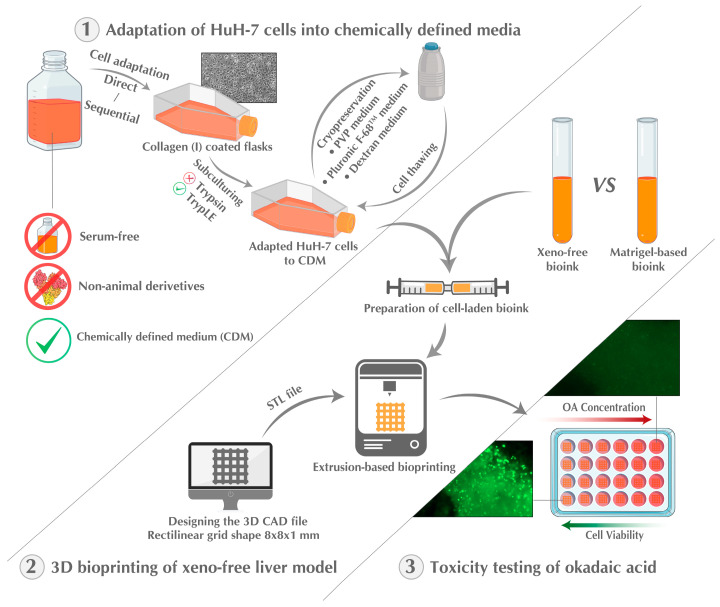
Schematic representation of the experimental design used in the study. Firstly, the HuH-7 cells were adapted to CDM using direct and sequential approaches. Next, freezing conditions were optimized, and a xeno-free liver model was developed using 3D bioprinting. Finally, the 3D bioprinted liver model was used to test the hepatotoxicity of okadaic acid.

The present study describes the development of a fully xeno-free 3D bioprinted liver model. As a demonstration of its utility, the hepatoxicity of okadaic acid is investigated using the model. The study’s approach includes the adaptation of HuH-7 cells to CDM by direct and gradual adaptation, the replacement of porcine trypsin during cell culture, the formulation of efficient cryopreservation freezing media, and the utilization of a xeno-free bioink for the 3D bioprinting of liver model by an extrusion printer (Figure 1). The xeno-free bioprinted liver models were used to investigate the hepatotoxicity of okadaic acid, a marine biotoxin generated by various types of dinoflagellates, in comparison with 2D cell culture. The study establishes the groundwork for forthcoming research endeavors and practical implementation in animal-free bioprinting. Additionally, it paves the path for further advancements in the research of animal-free bioinks and adaptation of various cell types to CDM.

## 2. Results

### 2.1. Adaptation of HuH-7 Cells to CDM

**Figure 2:**
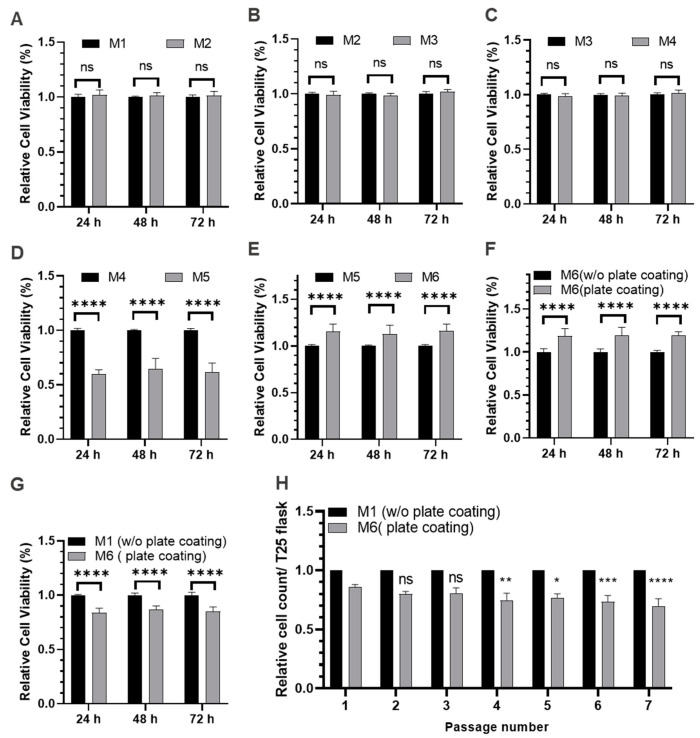
Viability of HuH-7 cells cultured directly in different media formulations to test the individual components. (**A**–**G**) Metabolic activity of HuH-7 cells cultured in different media formulations was determined by the XTT assay. Mean of data in black columns were used as reference to calculate the relative cell viability at each corresponding time point. (**H**) Relative cell counts for successive cell passages. Mean of data in black columns were used as reference to calculate the relative cell count at each corresponding passage number. Data are presented as the mean ± standard deviation; *n* = 3. ns *p* > 0.05, * *p* < 0.05, ** *p* < 0.01, *** *p* < 0.001, and **** *p* < 0.0001. Data in black columns were used as reference to calculate the relative cell viability at each time point. The relative cell count of successive cell passages are calculated as a percentage of the mean of cell count of the data presented in black columns for each passage number.

Two approaches were employed for the adaptation of HuH-7 cells to CDM: direct adaptation and gradual adaptation. Direct adaptation was used as a preliminary strategy to rapidly assess the individual components of the CDM, while gradual adaptation, which involves successive subculturing, was undertaken to ensure the smooth adaptation of cells without extreme reduction in FBS. The compositions of media used for direct adaptation are listed in Table 1. HuH-7 cells, initially cultured in 10% FBS-containing DMEM low glucose, were transferred to a 50:50 mixture medium of DMEM and Ham’s F-12 basal medium supplemented with 10% FBS. Cell viability was assessed after 24, 48, and 72 h using the XTT assay. Notably, HuH-7 cells exhibited a favorable response to the DMEM/F12 medium, as indicated by consistent high viability levels (Figure 2A). Based on these results, DMEM/F12 was selected to replace DMEM low glucose in subsequent experiments. Data are presented as calculated as a percentage of the mean of cell count of the data presented black columns at each time point.

L-glutamine, essential for cell growth and viability, degrades over time under culture conditions. To address this, GlutaMAX, a stable alternative that releases L-glutamine as needed, was incorporated into the culture medium at a final concentration of 2 mM. Viability comparisons between media with GlutaMAX and L-glutamine demonstrated comparable results after 24, 48, and 72 h (Figure 2B). As adaptation is a long-term process, antibiotics are generally included to minimize the risk of bacterial contamination, although their potential impact on cell viability under these conditions has not been tested directly. We therefore investigated the impact of penicillin/streptomycin (P/S) on cell viability. Viability assessments at 24, 48, and 72 h indicated that the presence of P/S had no noticeable effect on the viability of HuH-7 cells (Figure 2C). We therefore included P/S in subsequent experiments to ensure protection against bacterial contamination.

When FBS was completely removed from the culture medium, HuH-7 cell viability was reduced by about half (Figure 2D) highlighting the importance of further improvement in the composition of the media. Epidermal growth factor (EGF) and hepatocyte growth factor (HGF) were incorporated into the culture medium at a concentration of 10 nM each. The inclusion of EGF and HGF led to noticeable improvements in cell viability (10–20%) (Figure 2E), indicating their positive influence on HuH-7 physiology.

Coating plastic culture plates with collagen is widely used to improve cell attachment and growth. This surface modification can be implemented to enhance charge and hydrophilicity on the polystyrol plastic surface. For research applications, collagen of porcine origin or from rat tails is commonly used. For the purpose of our study, collagen type I extracted from human placenta was used. As depicted in Figure 2F, collagen coating of the plate had a positive impact on cell viability.

As a final analysis, cell growth under conventional conditions in FBS-containing M1 medium and under optimized conditions in FBS-free M6 medium on pre-coated plates was compared. The average cell viability of cell cultured under FBS-free conditions ranged between 85% and 90% in comparison to those cultured under conventional conditions with FBS (Figure 2G). For HuH-7 cells grown in CDM M6, no morphological differences became obvious compared to those grown in the standard medium M1 containing 10% FBS (Figure S2). To validate the durability of the FBS-free culture conditions, cells were sequentially subcultured for seven passages with a 1:2 split ratio. Over the course of this experiment, we observed only a slight (albeit statistically significant) decrease in cell viability of approximately 10% (Figure 2H).

**Figure 3:**
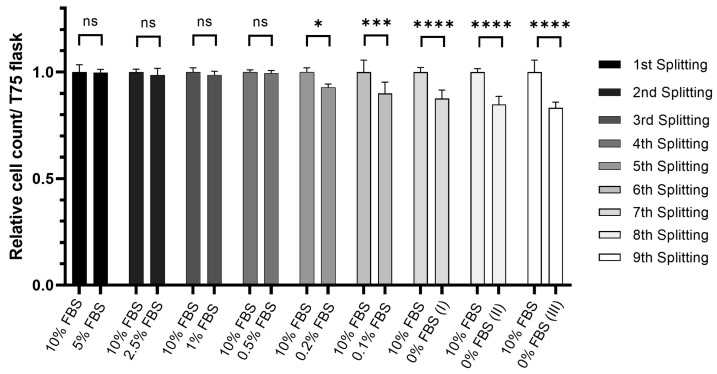
Sequential adaptation of HuH-7 cells to CDM. Relative cell count of HuH-7 cells cultured at sequentially reduced concentrations of FBS in the media. The mean of data from 10% FBS cell culture was used as a reference to calculate the relative cell count at each corresponding splitting; *n* = 3. ns *p* > 0.05, * *p* < 0.05, *** *p* < 0.001, **** *p* < 0.0001.

In the gradual adaptation, the cell count was monitored across successive passages. Initially, cells were cultured in M1 media with 10% FBS. For the gradual adaptation, M6 medium and coated plates with varying concentrations of FBS were used. In the initial step, the FBS content was reduced to 5% without noticeable changes in the cell count (Figure 3). Likewise, cell count remained high when the FBS content was reduced successively down to 0.5% FBS. This finding indicates that cells can take up all the required nutrients from 10–20-fold lower amounts of FBS compared to standard conditions without remarkable changes in their proliferation rate. As the FBS concentration was further decreased in subsequent passages, the relative cell count exhibited a gradual decline. At 0.2% FBS, a small but statistically significant decline in cell proliferation was observed, a trend that became more pronounced upon further reduction of the FBS content. When FBS was completely eliminated from the culture medium (0% FBS), the relative cell count decreased to approximately 85% in comparison to the M1 culture.

As changes in the culture conditions may have a substantial impact on cell physiology, it is crucial to analyze possible effects of the adaptation procedure on cell morphology. As can be seen in the phase contrast images shown in Figure S1A, no substantial changes in cell morphology occurred during the adaptation process.

Interestingly, we did not observe substantial differences between the direct and gradual adaptation strategy. For a comparative experiment, HuH-7 cells initially cultured in M1 medium were concurrently transferred directly to M6 culture medium. In parallel, cells were gradually adapted by continuous reduction of the FBS content until the medium was completely depleted of FBS. As depicted in supplementary Figure S1B, the relative cell count remained consistent between the direct and gradual adaptation across three consecutive passages.

Taken together, the adaptation experiments suggest that HuH-7 cells can be grown at substantially (10–20-fold) reduced FBS content compared to standard conditions without noticeable decrease in cell proliferation. For further reduction of the FBS content or for cultivation in media completely free of FBS, additional growth factors and coating of the cell culture plates is necessary. For all further experiments, the HuH-7 cells gradually adapted to FBS-free M6 medium were used.

### 2.2. Cell Detachment without Animal-Derived Enzymes

**Figure 4:**
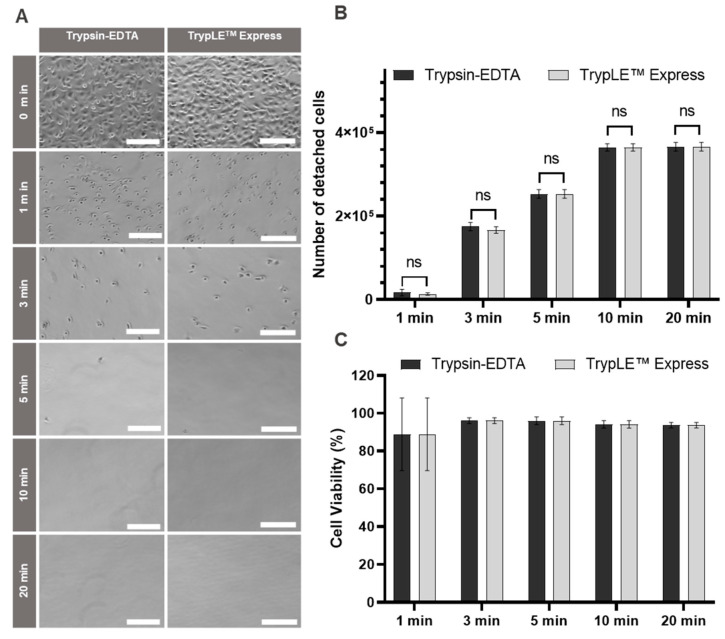
Detachment of HuH-7 cells by Trypsin-EDTA and TrypLE^TM^ Express. (**A**) Representative optical microscope images of HuH-7 cells treated with Trypsin-EDTA and TrypLE Express at the indicated time points. Scale bar: 200 μm. (**B**) Absolute number of detached HuH-7 cells after treatment with Trypsin-EDTA and TrypLE Express at the indicated time points; *n* = 3. ns *p* > 0.05. (**C**) Cell viability of HuH-7 cells after treatment with Trypsin-EDTA and TrypLE Express after the indicated incubation times. The values were calculated based on counting with a Neubauer counting chamber after staining with trypan blue.

Standard cell culture methods include detachment of the cells from culture dishes with trypsin, which is commonly isolated from porcine pancreas [46]. Alternative products that are not of animal original have become commercially available, e. g., recombinant fungal trypsin-like protease. To eliminate animal-derived products from cell culture procedures, we compared detachment by conventional trypsin and animal-free TrypLE^TM^ Express. As can be seen in Figure 4A, cell detachment was rapid with both proteases, and the few remaining undetached cells gained a rounded morphology even after only 1 min of treatment. After 5 min of treatment, hardly any cells remained on the cell culture flask, as can be seen in the microscopic images. Virtually no differences were observed for the numbers of cells detached with Trypsin-EDTA and TrypLE (Figure 4B). Likewise, cell viability was high for all detachment periods under investigation (up to 20 min), regardless of the protease used (Figure 4C). Taken together, animal-free TrypLE performed equally well as animal-derived trypsin. As both enzymes are equal in cost, it is easy to replace this animal-derived material in standard cell culture procedures.

### 2.3. Cryopreservation

**Figure 5:**
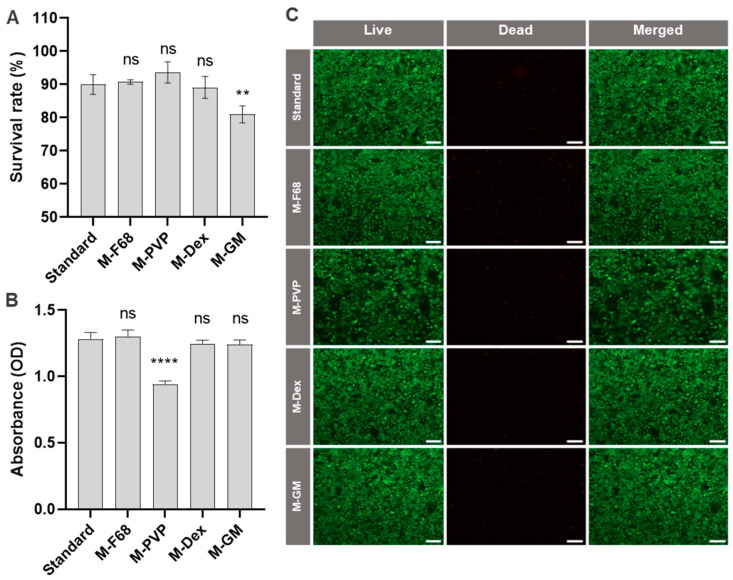
Cryopreservation of HuH-7 cells in different freezing media. (**A**) Survival rate of HuH-7 cells after freezing for 7 days. The values were calculated based on cell counting with a hemocytometer after staining with trypan blue; *n* = 3. ns *p* > 0.05, ** *p* < 0.01 compared to standard. (**B**) HuH-7 cells were cryopreserved in the indicated freezing media. After thawing, they were cultured for 48 h before metabolic activity was determined by the XTT assay; *n* = 3. ns *p* > 0.05, **** *p* < 0.0001. (**C**) Fluorescence microscopic analysis of cell viability by live/dead staining 48 h after thawing from indicated freezing media. Cells were stained with calcein-AM (live in green) and ethidium homodimer-1 (dead in red). Scale bar: 200 μm.

Another important procedure used in mammalian cell culture is the freezing of the cells in a cryoprotective medium. For this step, a freezing medium composed of 90% FBS with 10% DMSO is commonly recommended [47]. We therefore aimed at replacing this medium with chemically defined freezing medium (CDFM). To this end, cells were frozen in various CDFM, and the cryopreserved cells were then thawed and subjected to quantitative assessment of survival rates using trypan blue staining. In addition to the standard medium, four alternative media were tested. In one mixture, the FBS was replaced by the culture medium M6. Cells frozen in this medium had the lowest viability after they were taken back into culture as determined by cell counting. The other CDFM were composed of 10% DMSO, the culture medium M6 and small amounts of dextran, PVP, and pluronic-F68, respectively. Interestingly, for all three CDFM, the cell viability of recultivated cells was similar to that of cells cryopreserved in the standard freezing medium (90% FBS and 10% DMSO). The medium containing PVP supported slightly (but statistically not significant) higher survival rates compared to the other media (Figure 5A).

To characterize the effects of freezing in CDFM in more detail, metabolic activity (Figure 5B) and cell viability (Figure 5C) were analyzed. Interestingly, the metabolic activity of cells that were frozen in CDFM with PVP was significantly lower compared to that of all other samples—i.e., the culture seemed to contain a number of viable cells that were counted by the hemocytometer but were metabolically inactive. Likewise, the density of viable green fluorescing cells of this culture in the live/dead assay was comparatively lower. Taken together, this systematic analysis revealed that common freezing medium containing 90% FBS can be replaced by CDFM while maintaining high cell viability and metabolic activity after taking cells back into culture.

### 2.4. Rheological Characterization of Bioinks

**Figure 6:**
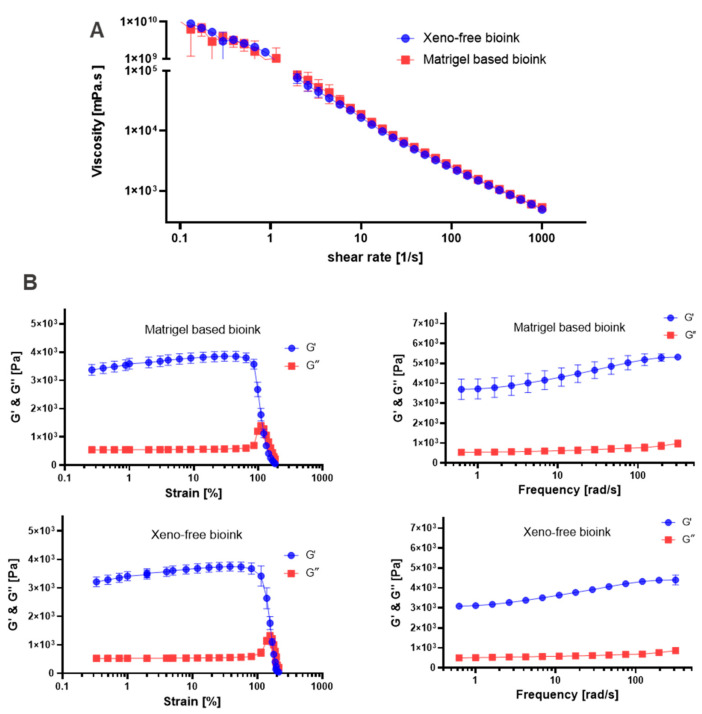
Rheological properties of crosslinked bioinks: (**A**) flow curve and (**B**) amplitude sweeping (**left side**) and frequency sweeping (**right side**).

So far, virtually all bioinks reported in the literature contain animal components, most commonly gelatin, gelatin methacryloyl (GelMa), collagen or BME. To prevent the generation of a chimeric system composed of human cells cultured in hydrogel containing animal components, we developed a xeno-free bioink, which we compared to a standard bioink containing Matrigel (Tables S4 and S5). Flow analysis was performed for both bioinks to assess their printability (Figure 6A,B). Both bioinks exhibited shear-thinning behavior, where the viscosity decreased as the shear rate increased. The viscosity range was comparable between the two bioinks. Specifically, the viscosity range for the Matrigel-based bioink was 1.04 × 10^10^ to 0.54 Pa∙s, while that for the xeno-free bioink ranged from 1.22 × 10^10^ to 0.311 Pa∙s. In the amplitude sweep experiment, both bioinks exhibited viscoelastic behavior, where their storage modulus (G′) was higher than their loss modulus (G″) (Figure 6B). This indicated that both bioinks displayed a solid-like nature within the tested range of frequencies. Notably, the Matrigel-based bioink demonstrated a slightly higher G′ (3587 Pa at 1% strain) compared to the xeno-free bioink (3411 Pa at 1% strain). Taken together, however, this rheological characterization demonstrates that the xeno-free bioink has very similar properties to those of the bioink containing Matrigel and can thus be expected to be well-suited to bioprinting approaches.

The frequency sweep test was conducted on both bioinks over a range of frequencies from 0.628 rad/s to 314 rad/s (Figure 6B). In both cases, the storage modulus was found to be consistently higher than the loss modulus. Furthermore, the increase in storage modulus observed in both bioinks as the frequency increased indicates that the materials become stiffer at higher frequencies. We noted that the Matrigel-based bioink exhibited a higher storage modulus (G′) in comparison to the xeno-free bioink across the frequency sweep. Nevertheless, the difference in G′ values can be considered minimal.

### 2.5. 3D Bioprinting of Xeno-Free Liver Model

**Figure 7:**
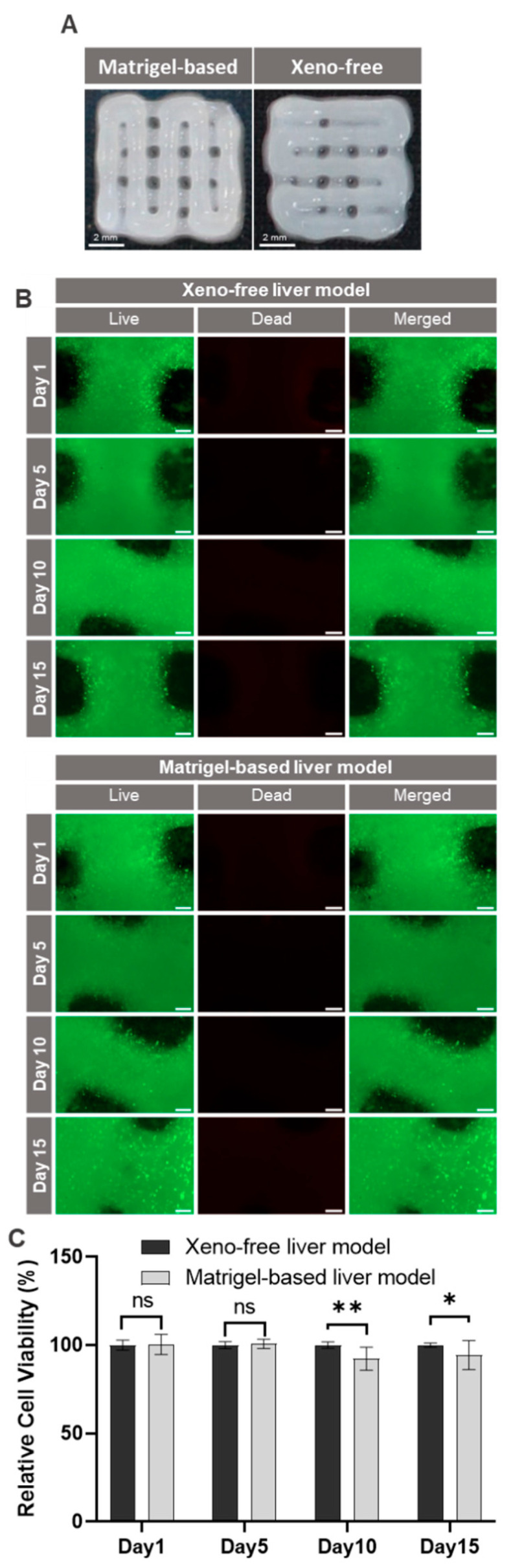
Viability of HuH-7 cells in 3D printed xeno-free and Matrigel-based liver models. (**A**) Images of printed grid constructs show the printability of the prepared bioinks (without cells). (**B**) Qualitative viability staining of living and dead HuH-7 cells in bioprinted models at the indicated time points stained with calcein-AM (live in green) and ethidium homodimer-1 (dead in red). Scale bar: 200 μm. (**C**) Metabolic activity of HuH-7 cells in the bioprinted liver models determined by XTT assay at the indicated time points. The mean of absorbance data from the xeno-free liver model was used as reference to calculate the relative cell viability at each corresponding time point; *n* = 3. ns *p* > 0.05, * *p* < 0.05, ** *p* < 0.01.

The main aim of the present study was the creation of a fully xeno-free liver model by 3D bioprinting. A commonly used grid model was printed with the xeno-free bioink, as well as with the standard bioink containing Matrigel. As can be seen in Figure 7A, both bioinks were suitable for producing the grid model, although the structural integrity of the model containing Matrigel was slightly better. Additionally, the printability of both bioinks was evaluated across two different printing batches, demonstrating favorable printing capabilities (Figure S4).

For the generation of the liver model, HuH-7 liver cells adapted to CDM were printed in a xeno-free hydrogel into a three-dimensional structure. Viability of the cells in the printed liver models was assessed up to 15 days in culture by live/dead staining. Figure S5 shows the reproducibility of bioprinting with good cell viability in both bioinks. The fluorescence images shown in Figure 7B do not reveal any noticeable difference between the models produced with the xeno-free bioink and those using the bioink containing Matrigel. Likewise, metabolic activity as determined by XTT assays was comparable for both bioinks, except that there was a very small, but statistically significant reduction in metabolic activity in the models containing Matrigel at later time points of the culture (Figure 7C). These experiments demonstrate that the fully xeno-free system can be used to create a 3D liver model by bioprinting which has a high long-term stability and metabolic activity and good structural integrity. All features were comparable to those of the model created with a bioink containing Matrigel.

### 2.6. Testing the Hepatotoxicity of Okadaic Acid

**Figure 8:**
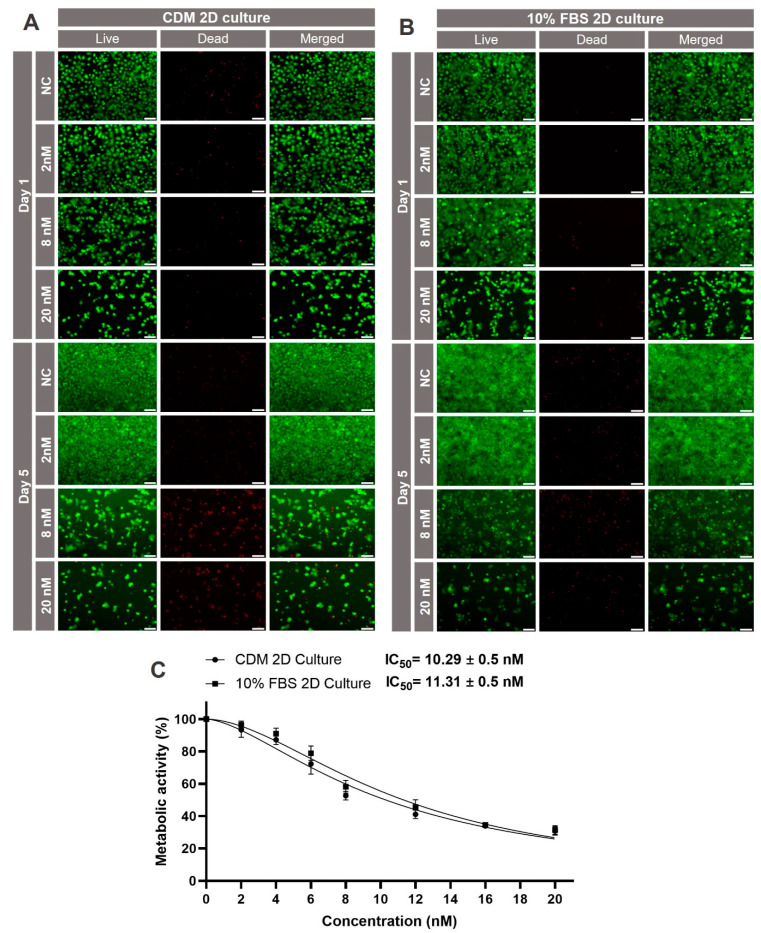
Okadaic acid-induced hepatoxicity in HuH-7 cells cultured in chemically defined or FBS-containing medium. (**A**,**B**) Qualitative viability staining of living and dead HuH-7 cells after treatment with the indicated concentrations of okadaic acid on day 1 and 5. Cells were stained with calcein-AM (live in green) and ethidium homodimer-1 (dead in red). Scale bar: 200 μm. (**C**) Metabolic activity of HuH-7 cells treated with the indicated concentrations of okadaic acid on day 5; *n* = 3.

The liver is the major organ metabolizing xenobiotics, and at the same time, its functionality is highly affected by exogenous toxins [48]. As an example, the hepatotoxicity of the marine biotoxin okadaic acid was investigated. OA is a potent and selective inhibitor of serine/threonine phosphatases 1 and 2A. It can induce rapid metabolic effects, potentially resulting in cell death by disrupting the balance of phosphorylation and dephosphorylation rates in vivo and in vitro. OA has previously been shown to affect the viability of human liver cells in vitro at nanomolar concentrations. Prior to testing toxicity in the 3D liver model, it was first investigated in 2D cell culture. Huh-7 cells cultured at a density of 1.5 × 10^4^/well in a 96 well-plate, either in standard medium with 10% FBS or in FBS-free CDM, were treated after 24 h of culture with increasing concentrations of okadaic acid for 5 days. Cell viability was assessed by live/dead staining on day 1 and 5 of the treatment. As can be seen in Figure 8A, cell viability was high on day one of the HuH-7 culture in CDM in the absence of okadaic acid. As expected, cell viability decreased with increasing concentrations of the toxin. The effects became even more pronounced after 5 days of culture. A similar trend was observed for the standard culture in the presence of 10% FBS.

To have a quantitative measure for the toxic effects of okadaic acid, XTT assays were performed on day 5 of the culture. All data were normalized to the untreated samples of each group. As can be seen in Figure 8B, metabolic activity of the HuH-7 cells decreased after treatment with okadaic acid in a concentration-dependent manner. Most importantly, the course of the toxicity curves was nearly identical for the standard culture in the presence of 10% FBS and for the culture in CDM. Very similar IC_50_ values of 10.2 ± 0.5 nM and 11.3 ± 0.5 nM, respectively, were calculated for the two conditions.

**Figure 9:**
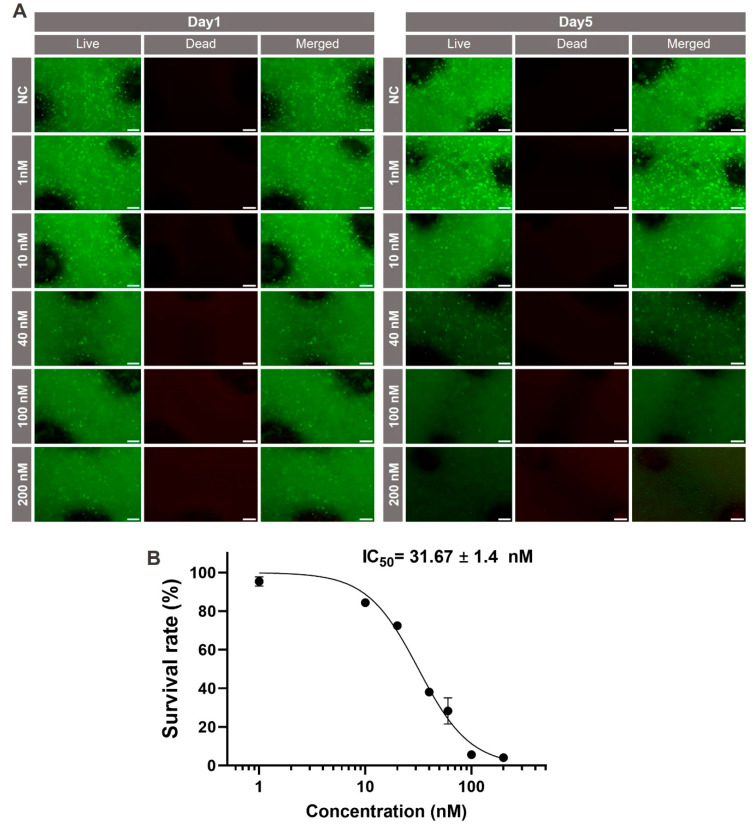
Viability of HuH-7 cells in 3D bioprinted xeno-free liver models after treatment with different concentrations of okadaic acid. (**A**) Qualitative viability staining of living and dead HuH-7 cells in the bioprinted models treated with the indicated concentrations of okadaic acid on day 1 and 5. Cells were stained with calcein-AM (live in green) and ethidium homodimer-1 (dead in red). Scale bar: 200 μm. (**B**) Metabolic activity of HuH-7 cells in the bioprinted models treated with the indicated concentrations of okadaic acid on 5; *n* = 3.

In the final experiment (Figure 9), the hepatotoxicity of okadaic acid was assessed in xeno-free 3D models. Live/dead assays and measurements of the metabolic activity were carried out on day 1 and 5 of the treatment. According to live/dead staining, the viability of HuH-7 cells remained stable up to a concentration of 10 nM okadaic acid on day 1. Only at concentration of 40 nM and above was a decrease in cell viability observed, as indicated by dimmer fluorescence. On day 5 of the treatment, the effects were much more pronounced (Figure 9A).

Again, XTT assays were performed to generate quantitative data for the hepatotoxicity of okadaic acid. As can be seen in Figure 9B, cytotoxicity became obvious at higher concentrations of the toxin than in 2D cultures, and an IC_50_ value of 31.7 ± 1.4 nM was calculated. This value is significantly higher than the ones calculated for the monolayer experiment. Altogether, these experiments demonstrate that the bioprinted xeno-free liver model can be used to study the hepatotoxicity of substances without using animal components at any point of the procedure. There is no proof that the mechanism of OA toxicity differs between 2D and 3D culture. However, diffusion of compounds might be altered between the different cultivation techniques. Another explanation for the different sensitivity of cells in 2D and 3D could be cultivation-dependent changes in drug efflux [49]. Another aspect might be drug metabolism, which may be changed under different cultivation conditions or dependent on cultivation time [50].

## 3. Discussion

Animal experimentation has played a significant role in advancing biomedical research, providing valuable approaches for studying disease mechanisms and toxicity testing. However, ethical concerns surrounding the welfare of animals and their experience of suffering has led to a growing awareness of the need to replace, reduce, and refine animal testing and to reduce the usage of animal components in biomedical research. Ethical concerns have sparked a re-evaluation of animal experimentation leading to the realization that toxicity testing in animals is of limited predictive value and is mainly justified by the lack of reasonable alternatives. Among a wealth of newly developed alternatives, 3D bioprinting has emerged as a new technique within this paradigm shift [5,51]. This technology allows for precise placement of cells, biomaterials, and bioinks, facilitating the construction of intricate three-dimensional biological structures [52]. Nonetheless, 3D bioprinting frequently involves the use of culture supplements and other materials sourced from animals [14]. The present study describes the development of a bioprinted liver model devoid of any animal constituents. The strategy includes the adaptation of HuH-7 cell to FBS-free media, cryopreservation, and the formulation of a xeno-free bioink for 3D bioprinting. To verify the quality of the xeno-free liver model, it was used for the assessment of okadaic acid-induced hepatotoxicity.

### 3.1. Adaptation of HuH-7 Cells to CDM

HuH-7 is a human liver cell line, which is widely used in virus research, oncology, and toxicity testing [46,53]. Basic protocols for mammalian cell culture recommend media supplemented with 10% FBS [47], which is also proposed for HuH-7 cells. The harvesting of FBS from unborn calves, however, has been criticized as being a cruel procedure and has been associated with the risk of pathogen transmission [16]. Furthermore, the supplementation of cell culture media with FBS generates unnatural chimeric systems of human cells in animal-derived sera and is associated with batch-to-batch variability that may hamper reproducibility [17]. In a previous study, HuH-7 cells were cultured in a serum-free medium supplemented with lipid-rich albumin [54]. However, the lipid rich albumin is an animal-derived product.

Therefore, the first task of our study was to adapt HuH-7 cells to a CDM free of animal-derived components. The adaptation process followed guidelines recommended in prior publications [22,55]. Two approaches, direct and gradual adaptation, were employed to facilitate adaptation. Direct adaptation allowed for a rapid assessment of the components within the CDM. The first step was the transfer of cells into DMEM/F12 medium, followed by minor changes including the introduction of GlutaMAX as a source for L-glutamine and the addition of antibiotics. However, when the serum was omitted, cell viability dropped to approximately one half. This observation proved that further additives that support cell growth were required to obtain sufficient amounts of cells in a reasonable time. Hepatocyte growth factor (HGF), also known as scatter factor (SF), is a protein that plays a crucial role in cell growth, tissue repair, and regeneration. It is primarily produced by mesenchymal cells and acts on various cell types, particularly hepatocytes, to stimulate cell proliferation, motility, and morphogenesis [56,57]. Epidermal growth factor (EGF) is another growth factor that is involved in the regulation of growth and differentiation of hepatocytes [58]. Therefore, EGF and HGF were added to the cell medium at a concentration of 10 nM each. Supplementation with EGF and HGF led to significant enhancement in cell viability of 10–20% (Figure 2E). Further improvement was achieved by coating the cell culture dishes to enhance cell attachment and growth by improving the charge and hydrophilicity of the plastic surface. To circumvent the need to include animal-derived collagen, which is widely used for this step, we coated the dishes with collagen type I extracted from human placenta. This step achieved a further increase in cell proliferation. We demonstrated that HuH-7 cells grow well in optimized FBS-free medium over multiple passages. This is important to show as cells may store some FBS components and grow well in FBS-free media for one or two passages but stop proliferating thereafter in suboptimal media. The observed slight reduction in cell proliferation of 10–15% over seven passages will be acceptable for most research applications. It may well be argued that the high proliferation rates triggered by FBS do not reproduce natural cell physiology in most cases.

To prevent possible cell damage due to the rapid depletion of FBS in direct adaptation, we carried out a gradual adaptation process with the optimized (M6) medium supplemented with decreasing concentrations of FBS. Interestingly, no substantial changes in the proliferation rate were observed during the adaptation process down to FBS concentrations as low as 0.5%. Many cell lines can be cultured at much lower concentrations than the standard 10% FBS, as, for example, reported for human mononuclear cells which were successfully cultured at 2–3% FBS [59].

Complete omission of FBS in the culture medium, however, requires the addition of further factors to support sufficient cell viability. Under these conditions, the cell count was reduced by approximately 15%, which might initially appear as a limitation of the cells adapted to CDM but can actually offer a significant advantage for prolonged toxicity testing, as previously documented in macrophage systems [60]. Importantly, cell morphology remained unaltered in our study upon depletion of FBS.

While the value of omitting FBS from mammalian cell culture has been apparent for a long time, very few studies have been carried out without any animal components in the media. This is due to the laborious and expensive process that has to be carried out for each individual cell line. In some cases, media from commercial sources are available; however, they are commonly too expensive for continuous use, at least in academic laboratories. In our study, we openly describe all details and the media composition for FBS-free cultivation of HuH-7 cells. We therefore hope that other groups involved in research with hepatic viruses or hepatotoxicity testing will adopt these culture conditions. In a recent publication, the Oredsson group published a general FBS-free medium with the potential to be widely applicable for normal and cancer cells [61]. Although the composition of this medium is rather complex, making it costly, it can advance attempts to decrease the use of FBS in mammalian cell culture.

### 3.2. Cell Detachment

Detachment of cells is a routine step in culturing adherent cells. Trypsin-EDTA is the most commonly used reagent for this procedure [47]; however, trypsin is usually of porcine origin, sparking ethical issues regarding its use in cell culture. Additionally, a previous study has highlighted potential alterations in the expression of surface antigens due to trypsin usage [62]. Therefore, we tested an alternative non-animal reagent, TrypLE Express against the conventional trypsin-EDTA. This comparison revealed that TrypLE is as efficient as the commonly used trypsin-EDTA and does not induce any alterations in cell viability. As the costs for both enzymes are comparable, TrypLE can easily be employed as an animal-free alternative to Trypsin-EDTA [62].

### 3.3. Cryopreservation

Cryopreservation is another important routine procedure in mammalian cell culture. A mixture of 90% FBS and 10% DMSO is commonly used as freezing medium to ensure high cell viability after thawing [47]. For the transition to FBS-free cell culture, it is important to identify freezing media without FBS for the adapted cell lines. For the HuH7 cells, we therefore tested various CDFM containing PVP, Dextran, or Pluronic F68. All tested freezing media exhibited remarkable cell survival rates, averaging around 90%, which is comparable to the level achieved with the conventional freezing medium.

To study the impact of the freeze/thaw procedure in different media on physiological function, metabolic assays were carried out after re-culturing of the cells. The metabolic activity of the cells remained stable across most of the tested freezing media, suggesting that the preservation process did not significantly impair their ability to regain metabolic function post-thaw. However, cells frozen in the PVP-based medium exhibited somewhat reduced metabolic activity upon re-culturing. This may reflect the fact that PVP is a polymer that may have a detrimental influence on cellular function. The observations from the metabolic activity assessment were corroborated by live/dead staining, which revealed lower cell density after freezing in CDFM containing PVP. Taken together, for the cryopreservation of FBS-free adapted HuH-7 cells, we recommend a CDFM composed of 85% M6 medium (Table 1) supplemented with 10% DMSO and 5% dextran or of 89% M6 medium supplemented with 10% DMSO and 1% Pluronic F68.

### 3.4. Rheological Characterization of Bioinks and Bioprinting of Xeno-Free Liver Model

Bioinks used for bioprinting approaches commonly contain animal components, such as gelatin, gelatin methacrolyl, collagen or BME [14]. Matrigel, the most popular commercially available BME product is derived from the Engelbreth-Holm-Swarm (EHS) mouse sarcoma. It is commonly used in bioink formulation for 3D bioprinting to provide a three-dimensional environment that mimics the natural extracellular matrix. However, the process of producing Matrigel involves introducing an EHS into mice, which raises severe ethical concerns [63]. Previous research by Baltazar et al. reported the development of a bioprinted xeno-free human skin graft based on xeno-free dermal and epidermal bioinks, where a commercial hydrogel was applied to minimize gel contraction and accelerate gelation [64]. In the current study, we aimed to develop a xeno-free liver model based on animal-free bioink with fully defined composition. The xeno-free bioink was formulated based on alginate and human collagen I, using CaSO_4_ as a cross-linking agent supplemented with amino acids and human serum as outlined in Table S5. For comparison, we used a typical bioink containing 20% Matrigel [11,65]. The exact compositions of both bioinks can be found in Tables S4 and S5. The xeno-free bioink is mainly based on human placental collagen and sodium alginate. Collagen is the main structural component of the ECM, exhibiting physicochemical properties that align with tissue requirements; therefore, it is extensively employed in various biomedical applications. Alginate is also a commonly used hydrogel with exceptional biocompatibility, its physical attributes can potentially be adjusted to guide 3D cell growth and differentiation [28]. To evaluate the rheological properties of the prepared bioinks, flow analysis was conducted. This analysis revealed that both bioinks exhibited shear-thinning behavior, a common phenomenon observed in viscoelastic materials where viscosity (η) decreases with increasing shear rate (γ). This behavior is crucial for successful 3D bioprinting, as it ensures that the bioinks can flow easily during the printing process, facilitating precise deposition [66]. Comparing the viscosity ranges of the two bioinks, we found them to be similar. This similarity in viscosity suggests that both bioinks can be effectively processed through the bioprinting nozzle without substantial differences.

We also carried out amplitude sweep experiments. The observed viscoelastic behavior of both bioinks was characterized by higher storage modulus (G′) than loss modulus (G″), which underscores their solid-like nature within the tested frequency range. This mechanical attribute is crucial for maintaining structural integrity and stability in the bioprinted constructs [67]. The subsequent frequency sweep test, spanning frequencies from 0.628 rad/s to 314 rad/s, further emphasized the solid-like behavior of both bioinks, with the storage modulus consistently exceeding the loss modulus. The observed increase in storage modulus with increasing frequency aligns with typical viscoelastic material responses [67]. Therefore, both bioinks exhibit promising characteristics for bioprinting according to their solid-like behavior and shear-thinning viscosity profiles.

The major experiment of the present study was the biofabrication of a tissue model with the xeno-free bioink and HuH-7 cells adapted to the CDM. For comparison, models were produced with the conventional bioink containing Matrigel. Both bioinks had good printability, and cell viability was very high in both models for the 15-day duration of the experiment. We thus conclude that the xeno-free system we developed is a suitable alternative to conventional human organ models, which contain various animal-derived components [68]. In the next step, we will aim at replacing the small amount of human serum used to supplement the bioink. While this does not interfere with the aim of producing a xeno-free bioink, the use of human serum may again result in batch-to-batch variability. Despite this point for further improvement, the model presented here fulfills the criteria of clean bioprinting, which we demanded in our previous review article [14], and thereby solves the ethical problems associated with the use of animal material as well as the scientific issues connected to chimeric systems composed of human and animal components.

### 3.5. Toxicity Testing

For proof-of-concept, the developed xeno-free model was used to evaluate the hepatotoxicity induced by okadaic acid in comparison to the conventional 2D cell culture. Okadaic acid is a naturally occurring marine toxin that is produced by certain species of dinoflagellates [69,70]. It is well-known for its potent inhibitory effects on protein phosphatases, particularly protein phosphatase 1 (PP1) and protein phosphatase 2A (PP2A) [71]. The hepatotoxicity of okadaic acid was examined in both 2D and 3D cultures of HuH-7 cells.

In the 2D culture, both the culture adapted to CDM and the one supplemented with 10% FBS exhibited concentration-dependent viability reduction with almost identical curves. The IC_50_ was calculated to be 10.2 ± 0.5 nM and 11.3 ± 0.5 nM for the adapted and 10% FBS-supplemented cultures, respectively. The 3D models displayed greater resistance to the substance, showing stable viability up to 10 nM on day 1, with a notable loss of cell viability at concentrations of 40 nM and above. By day 5, viability declined significantly even at 10 nM, with pronounced decreases at 100 nM and 200 nM. XTT assay results corroborated the live/dead staining findings, with an IC_50_ value of 31.7 ± 1.4 nM for the xeno-free 3D model on day 5. Higher resistance against cytotoxicity of 3D bioprinted models compared to 2D cultures has been reported by a number of groups, including ours, for various models and drugs [72,73,74,75]. This finding is most likely due to the cells interacting in 3D networks compared to monolayer cultures, in which all cells have direct contact with the medium with their whole surface all the time. Further research will help to elucidate the underlying mechanisms.

To improve liver physiology, it will be necessary to include further cell types such as sinusoid cells, macrophages, and endothelial cells in the liver model. While this is a great challenge for conventional models, it becomes even more demanding for xeno-free models, as each of these lines needs to be adapted to FBS-free CDM. Another hurdle for multi-cell type models is that a medium needs to be identified which supports growth of all cells in the co-culture.

We believe that the implementation of xeno-free bioprinting together with the other NAMs for risk assessment will significantly boost the generation and interpretation of data, thereby minimizing overall uncertainty in chemical safety evaluations. Nevertheless, to achieve this, it is essential to establish clear guidelines specifying the criteria for adopting these methodologies. This includes defining validation processes, implementing quality control measures, and standardizing reporting formats.

## 4. Materials and Methods

### 4.1. Standard Cell Culture

HuH-7 cells (330156, CLS Cell Lines Service GmbH, Eppelheim, Germany) were cultured in DMEM low glucose medium (L0064, Biowest, Nuaillé, France) with 10% fetal calf serum (FCS, S-14-L, c.c.pro GmbH, Oberdorla, Germany) supplemented with D-(+)-glucose (G8769, Sigma, Saint Louis, MO, USA) to a final concentration of 4.5 g/L, 2 mM L-glutamine (X0550, Biowest) and 1× penicillin/streptomycin (P/S, L0022, Biowest). Cells were cultured in a humidified incubator at 37 °C adjusted to 5% CO_2_. For subculturing, once cell confluency reached 70–80%, cells were washed twice with Dulbecco’s phosphate buffered saline (DPBS, L0615, Biowest) before harvesting. See below for cell detachment cell culture.

### 4.2. Coating of Cell Culture Vessels with Human Collagen Type I

All plates and vessels designated for 2D cell culture in CDM were pre-coated with human placental collagen I (THT0102, THT Biomaterials GmbH, Vienna, Austria). The lyophilized collagen I was dissolved in 0.25% acetic acid (27225-M, Sigma, Steinheim, Germany) under aseptic conditions to give a concentration of 3.2 mg/mL. Subsequently, the solution was diluted 1:100 in sterile H_2_O for direct coating or storage at −20 °C. The flasks and plates were coated with the diluted solution using the volumes specified in Table S1 and then incubated at 37 °C for 3 h, which allowed the collagen to adhere effectively. Once the coating process was complete, any excess solution was drained off, and the plates were thoroughly dried at room temperature. After coating, the plates were either used immediately or kept at 4 °C until they were utilized.

### 4.3. Adaptation of HuH-7 Cells to Chemically Defined Media (CDM)

The adaptation of HuH-7 cells to CDM was performed according to standard procedures [22,55]. Two schemes were used for the adaptation process: direct adaptation and gradual adaptation. In both schemes, the cryopreserved HuH-7 cells were retrieved and cultured in FBS-containing DMEM low glucose for two passages before starting the adaptation process. In the direct approach, cells were directly cultured in different formulations of DMEM-F12 (L0090, Biowest) with L-glutamine (X0550, Biowest), HEPES (4-(2-Hydroxyethyl)-piperazin-1-ethansulfonsäure, L0180, Biowest), 1× P/S (L0022, Biowest), FCS (S-14-L, c.c.pro GmbH), non-essential amino acids (NEAA, Biowest), GlutaMAX (35050, Gibco, Life Technologies limited, Paisley, UK) Insulin-Transferrin-Selenium (ITS, 41400045, Gibco, Life Technologies Corp, Grand Island, NY, USA), recombinant human hepatocyte growth factor (HGF, PHG0254, Gibco, Life Technologies Corp, Frederick, MD, USA), or recombinant human epidermal growth factor (EGF, PHG0313, Gibco, Life Technologies Corp, Carlsbad, CA, USA). The Composition of each medium is shown in Table 1. Cells were seeded in a pre-coated 24-well plate (92024, TPP Techno Plastic Products, Trasadingen, Switzerland) at a density of 1 × 10^5^ cells/well in the specified medium for 24, 48 and 72 h, respectively.

In the gradual adaptation, the amount of FBS in the culture medium was reduced in a stepwise manner. Cells were initially cultured under optimal conditions achieved through direct adaptation (M6 in Table 1), except for the FBS concentration which was 5%. Subsequently, cells were subcultured sequentially at decreasing FBS concentrations: 2.5%, 1%, 0.5%, 0.2%, 0.1%, and eventually 0% (*v*/*v*) FBS. Following the transfer to a fully CDM, cells were subcultured in a blend of 1 part conditioned medium and 4 parts fresh medium. The conditioned medium refers to the medium harvested from the previous cell culture. Details of cell line and cell culture reagents are listed in Tables S2 and S3.

### 4.4. Detachment of Cells from Culture Vessels

For comparative analysis, HuH-7 cells were cultured in pre-coated 12-well plates (2 × 10^5^ cells/well) for 48 h. Subsequently, cells were detached using trypsin-EDTA 1× (porcine-derived) or TrypLE^TM^ Express (animal-free, 12604021, Gibco, Life Science Corp., Grand Island, NY, USA) for 1, 3, 5, 10, and 20 min at 37 °C. Total cell count was determined using a Neubauer counting chamber, and undetached cells were imaged using an inverted fluorescence microscope (Zeiss Observer. Z1 microscope, Zeiss, Göttingen, Germany).

### 4.5. Freezing/Thawing Procedure

Different CDFM were prepared for cryopreservation of the adapted HuH-7 cells. The composition of each formula is shown in Figure S3. PVP (PVP360, Sigma, Steinheim, Germany), Dextran (92191, Roth), Pluronic F68™ (A1288, Applichem, Darmstadt, Germany) work as cryoprotectants. As control formulations, 90% FBS with 10% DMSO (D2650, Sigma, Steinheim, Germany) and 90% CDM with 10% DMSO were used. HuH-7 cells were cultured until they reached a confluence of 70–80%. Subsequently, cells were harvested using TrypLE and quantified before being centrifuged at 300× *g* for 3 min. The resulting cell pellets were then resuspended in ice-cold freezing media at a density of 1.5 × 10^6^ cells/mL. Next, 1 mL of the cell suspension was transferred to a Simport cryo-vial (E309, Roth). The NALGENETM cryo freezing container controlled a gradual cooling process at a rate of −1 °C/min until reaching −80 °C. Following a 24-h period at −80 °C, the samples were relocated to a liquid nitrogen tank for an additional 6 days before thawing. For retrieving cells from liquid nitrogen, the cryovials were incubated at 37 °C for 1–2 min in a water bath. Subsequently, the cells were resuspended and centrifuged at 300× *g* for 3 min. The cell pellets were resuspended in CDM and cultured in collagen-precoated T25 cell culture flasks.

### 4.6. Cell Viability

Cell viability assays by life/dead staining were performed for 3D and 2D cultures using ethidium homodimer-1 (E1169, Invitrogen, Eugene, OR, USA) that labels dead cells and calcein AM (800112, Biotium, Fremont, CA, USA) that labels viable cells. In brief, the bioprinted constructs underwent a 30-min incubation with phenol red-free RPMI (L0505, Biowest) supplemented with 2 µM calcein AM and 2 µM ethidium homodimer-1 after removing the supernatant and washing with PBS. Subsequently, fluorescence microscopy (Zeiss Observer. Z1 microscope) was used to analyze the signals, with green indicating live cells and red indicating dead cells. In the case of 2D cell culture, calcein AM and ethidium homodimer-1 were utilized at a concentration of 1 µM, using the same experimental conditions.

The metabolic activity in both 2D and 3D cell cultures was evaluated by XTT assays (2,3-bis-(2-methoxy-4-nitro-5-sulfophenyl)-2H-tetrazolium-5-carboxanilide, J61726, Alfa Aesar, Ward Hill, MA, USA) at the indicated time points according to the manufacturer’s instructions. A solution of 3.83 mg/mL phenazine methosulfate (PMS, A2212005, AppliChem, Darmstadt, Germany) and 1 mg/mL XTT salt was prepared at volume ratio of 1:500 for PMS and XTT salt subsequently. After a 4-h incubation period, absorbance intensity was measured at 450 nm with 620 nm as a reference wavelength using a microplate reader (Sunrise, Tecan, Männedorf, Switzerland). As a background control, a sample treated with 70% ethanol for 10 min was used to normalize all the absorbance values.

### 4.7. Bioink Formulation

Two different bioinks, a xeno-free bioink and a bioink containing Matrigel™, were prepared with the compositions depicted in Table S4. Firstly, a 10% alginate solution (W201502, Sigma) in sterile H_2_O was prepared. To ensure complete dissolution, the solution was continuously stirred at 37 °C overnight. Additionally, two supplement mixtures (Sup mix I and Sup mix II) were prepared as outlined in Table S5; human serum (H5667, Sigma, Taufkirchen, Germany), NEAA, P/S, GlutaMAX, and HEPES ensured the provision of essential nutrients to the cells during the critical initial hours after printing. Subsequently, 750 µL of the alginate solution was loaded into a 3 mL syringe, while 1200 µL supplement mixture, 150 µL 1 M CaSO_4_ (0256.2, Roth, Karlsruhe, Germany), 300 µL 3.2 mg/mL human collagen and 600 µL RPMI medium combined with 6 × 10^7^ HuH-7 cells were loaded in a separate 3 mL syringe (Cell density: 2 × 10^7^ /mL). Subsequently, the two syringes were connected using a female Luer coupler (45508-22, Masterflex, Gelsenkirchen, Germany), and the bioink was mixed at room temperature for 15 min directly before printing.

### 4.8. Rheological Measurements

The rheological properties of the bioink were measured using a rotational rheometer (Modular Compact Rheometer, MCR 102, Anton Paar, Ostfildern, Germany). Prior to analysis, the bioink was prepared according to the previously mentioned procedures without including cells in the bioink, then mixed for 20 min before starting the measurements. All measurements were recorded at 37 °C to mimic the bioprinting conditions. The viscosities and shear stress of the two bioinks were assessed with flow curves in a range of 0.1 to 1000 s^−1^ shear strain rate, allowing for the determination of the bioink’s shear-thinning behavior. Subsequently, a frequency sweep test was conducted to determine the linear viscoelastic region (LVR) of the bioink. The frequency sweep was performed over a frequency range of 0.628–314 rad/s with a constant strain amplitude within the LVR 1%. The amplitude sweep test was conducted at constant frequency. The strain amplitude gradually increased from 0.0001% to 1% in logarithmic increments.

### 4.9. Bioprinting

The bioink was extruded using a 21G nozzle at a pressure of 20–25 kPa using the BioX microextrusion printer (Cellink, Gothenburg, Sweden). The 3D construct, shaped like a grid and measuring 8 × 8 × 1 mm, was designed using the open-source software FreeCAD 0.20. To maintain optimal conditions during the printing process, the cartridge temperature was set to 37 °C. After printing, the constructs underwent post-treatment by submerging in 20 mM CaCl_2_ (5239, Roth) to ensure the complete gelation of alginate. Subsequently, the models were transferred to a new 24-well plate and cultured in CDM supplemented with 10 mM CaCl_2_ at 37 °C with 5% CO_2_. Media changes were performed every 48 h throughout the culture period. Details of used lab equipment for printing and characterization are listed in Table S6.

### 4.10. Statistical Analysis

Experimental data were collected for three independent replicates, and the results were presented as the mean ± standard deviation (SD) unless otherwise stated. Statistical analysis was performed using Prism 9 (Graphpad, Boston, MA, USA). One-way analysis of variance (ANOVA) was employed to compare groups. The significance level was set at * *p* < 0.05, ** *p* < 0.01, *** *p* < 0.001, and **** *p* < 0.0001 to determine statistical significance.

## 5. Conclusions

In conclusion, this study describes the development of a xeno-free 3D bioprinted liver model. The HuH-7 cells adapted to chemically defined media may be of general interest for liver research. In addition, the bioink formulation used here demonstrates that it is possible to create 3D tissue models devoid of animal components. The xeno-free model not only maintains high cell viability and metabolic activity on a par with traditional methods but can also be used to study hepatotoxicity. This advancement marks a step towards ethically sound and human-relevant substance screening approaches, holding great promise for safer and more effective drug development.

## Figures and Tables

**Table 1:** Composition of media used for direct adaptation.

Component	M1	M2	M3	M4	M5	M6
Basal Media	DMEM (Low glucose)	DMEM/F-12	DMEM/F-12	DMEM/F-12	DMEM/F-12	DMEM/F-12
L-glutamine	2 mM	2 mM	N/A	N/A	N/A	N/A
HEPES	N/A	10 mM	10 mM	10 mM	10 mM	10 mM
D-(+)-glucose	4.5 g/L	4.5 g/L	4.5 g/L	4.5 g/L	4.5 g/L	4.5 g/L
GlutaMAX	N/A	N/A	2 mM	2 mM	2 mM	2 mM
Penicillin/Streptomycin	N/A	N/A	N/A	1×	1×	1×
Fetal Bovine Serum	10%	10%	10%	10%	N/A	N/A
Non-essential amino acids	N/A	N/A	N/A	N/A	1×	1×
Insulin-Transferrin-Selenium	N/A	N/A	N/A	N/A	1×	1×
Hepatocyte growth factor	N/A	N/A	N/A	N/A	N/A	10 nM
Epidermal growth factor	N/A	N/A	N/A	N/A	N/A	10 nM

## Data Availability

Data are available upon reasonable request.

## Supplementary Materials

The following supporting information can be downloaded at: https://www.mdpi.com/article/10.3390/ijms25031811/s1.
