# Supplementary material for: Xeno-Free 3D Bioprinted Liver Model for Hepatotoxicity Assessment
Source: Int J Mol Sci. 2024 Feb 2;25(3):1811. doi: 10.3390/ijms25031811 (PMC10855587; doi:10.3390/ijms25031811)
Supplement: Supplementary file 1 [file ijms-25-01811-s001.zip › ijms-2819620-supplementary.pdf]

Supporting Material

Xeno-Free 3D Bioprinted Liver Model for Hepatotoxicity Assessment

Authors

*Ahmed S. M. Ali[1], Johanna Berg[1], Viola Roehrs[1], Dongwei Wu[1], Johannes Hackethal[2], Albert Braeuning[3], Lisa Woelken, Cornelia Rauh[4], Jens Kurreck[1, *]*

Affiliations

[1] Department of Applied Biochemistry, Technische Universität Berlin, Berlin, Germany
[2] THT Biomaterials, Vienna, Austria
[3] Department Food Safety, German Federal Institute for Risk Assessment (BfR), Berlin, Germany
[4] Department of Food Biotechnology and Food Process Engineering, Technische Universität Berlin, Germany

***Corresponding author**:
Prof. Dr. Jens Kurreck
Technische Universität Berlin, Institute of Biotechnology
Department of Applied Biochemistry, TIB 4/3-2
Gustav-Meyer-Allee 25, 13355 Berlin, Germany
Email: jens.kurreck@tu-berlin.de

Table S1. Volumes used to coat flasks and plates.

Vessel	Volume of diluted collagen I
T75 flask	5 ml per flask
T25 flask	2 ml per flask
12 well plates	500 µl per well
24 well plates	300 µl per well
96 well plates	50 µl per well

Table S2. Cell lines

Cell line	Catalogue number	Supplier
HUH-7	330156	CLS Cell Lines Service GmbH

Table S3. Chemicals and reagents

Item	Catalogue number	Supplier
DMEM low glucose medium	L0064	Biowest
Fetal calf serum (FCS)	S-14-L	c.c.pro GmbH
D-(+)-glucose	G8769	Sigma
L-glutamine	X0550	Biowest
Penicillin/streptomycin (100x)	L0022	Biowest
Dulbecco's phosphate buffered saline (DPBS)	L0615	Biowest
Human placental collagen I	THT0102	THT Biomaterials GmbH
Acetic acid	27225-M	Sigma
DMEM-F12	L0090	Biowest
4-(2-Hydroxyethyl)-piperazin-1-ethansulfonsäure (HEPES)	L0180	Biowest
MEM Non-Essential Amino Acids 100X (NEAA)	X0557 - 100	Biowest
GlutaMAX 100x)	35050	Gibco, Life Technologies limited
Insulin-Transferrin-Selenium (100x)	41400045	Gibco, Life Technologies Corp.
Recombinant human hepatocyte growth factor (HGF)	PHG0254	Gibco, Life Technologies Corp.
Recombinant human epidermal growth factor (EGF)	PHG0313	Gibco, Life Technologies Corp.
TrypLE™ Express	12604021	Gibco, Life Science Corp.
Polyvinylpyrrolidone (PVP)	PVP360	Sigma
2,3-bis-(2-methoxy-4-nitro-5-sulfophenyl)-2H-tetrazolium-5-carboxanilide (XTT)	J61726	Alfa Aesar
Phenazine methyl sulfate (PMS)	A2212,005	AppliChem
Sodium alginate	W201502	Sigma
Human serum	H5667	Sigma
Calcium sulphate dihydrate	0256.2	Roth

Table S4. Composition of bioinks

Component (each 1 mL)	Xeno-free bioink	Matrigel based bioink
RPMI	200 µL	100 µL
Sod. alginate (10%)	250 µL	250 µL
Human collagen I (3.2 mg/mL)	100 µL	N/A
$CaSO_4$ (1.22 M)	50 µL	50 µL
Matrigel	N/A	200 µL
Supplement mix (I)	N/A	400 µL
Supplement mix (II)	400 µL	N/A

Table S5. Composition of the supplement mixtures

Component (each 1 mL)	Supplement Mix (I) 2,5X	Supplement Mix (II) 2,5X
RPMI	850 µL	600 µL
Nonessential amino acids (NEAA)	25 µL	25 µL
HEPES (1M)	75 µL	75 µL
Penicillin – Streptomycin (10 000 000 U/L)	25 µL	25 µL
GlutaMAX	25 µL	25 µL
Human serum	0	250 µL

Table S6. Lab equipment

Equipment	Model	Supplier
Microextrusion printer	BioX	Cellink
Rotational rheometer	MCR 102	Anton Paar
Microplate reader	Sunrise	Tecan
Fluorescence microscope	Zeiss Observer. Z1	Zeiss

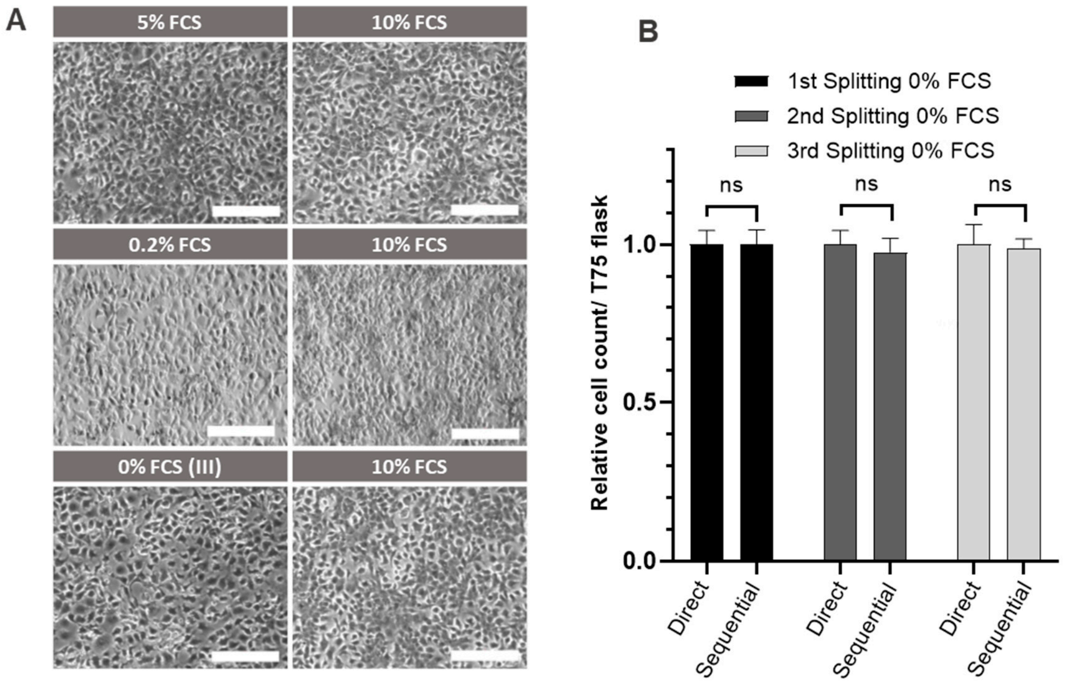

Figure S1. Sequential and direct adaptation of HuH-7 cells into CDM. (A) Representative optical microscope images in phase contrast mode of HuH-7 cells during sequential adaptation to CDM compared to that cultured in 10% FBS-containing media. Scale bar: 200 µm. (B) Relative cell count of sequentially pre-adapted HuH-7 cells in CDM compared to directly adapted cells.

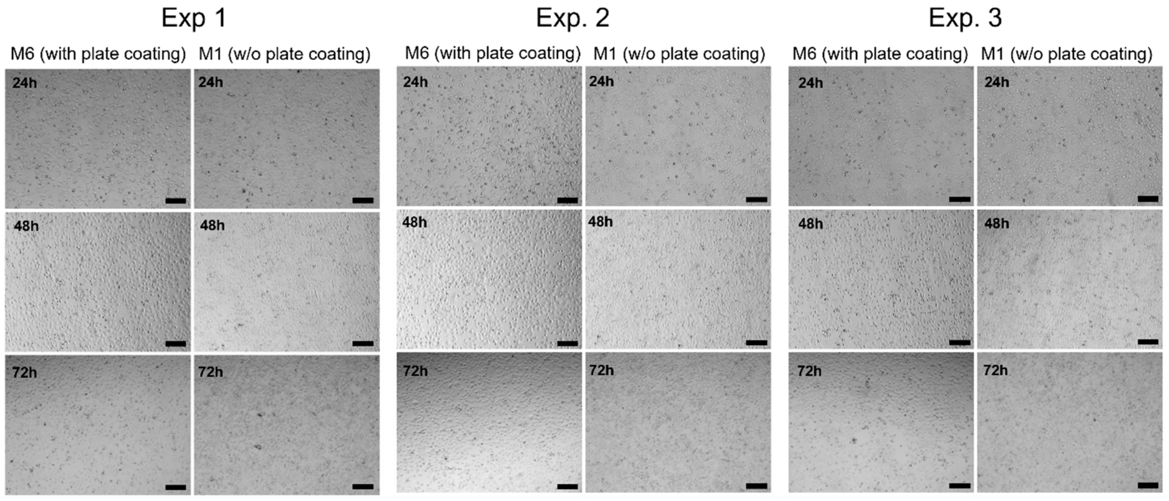

Figure S2. Representative optical microscope images of HuH-7 cells cultured in M6 (with plate coating) and M6 (without plate coating) after 24h, 48h and 72 h in three independent experiments. Scale bar: 200 µm.

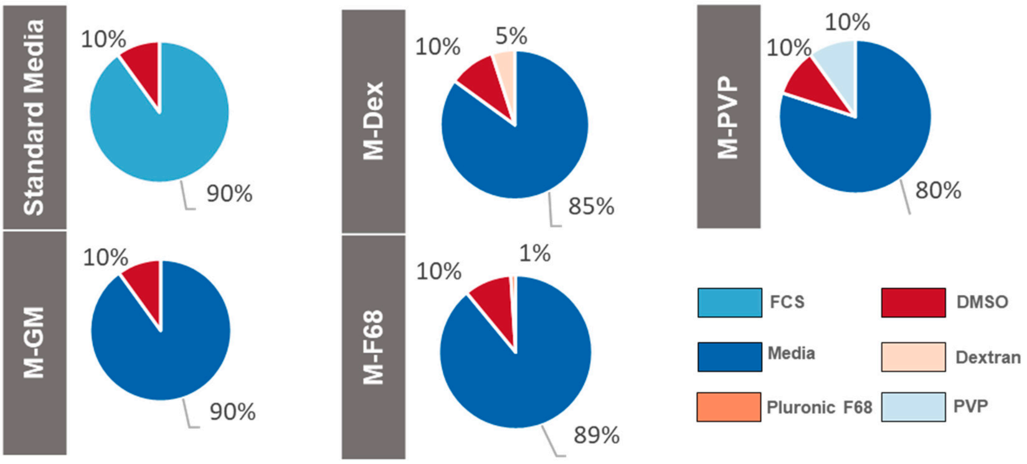

Figure S3. composition of the tested freezing media presented in pie charts. Each component is represented by a color as indicated in the lower right part.

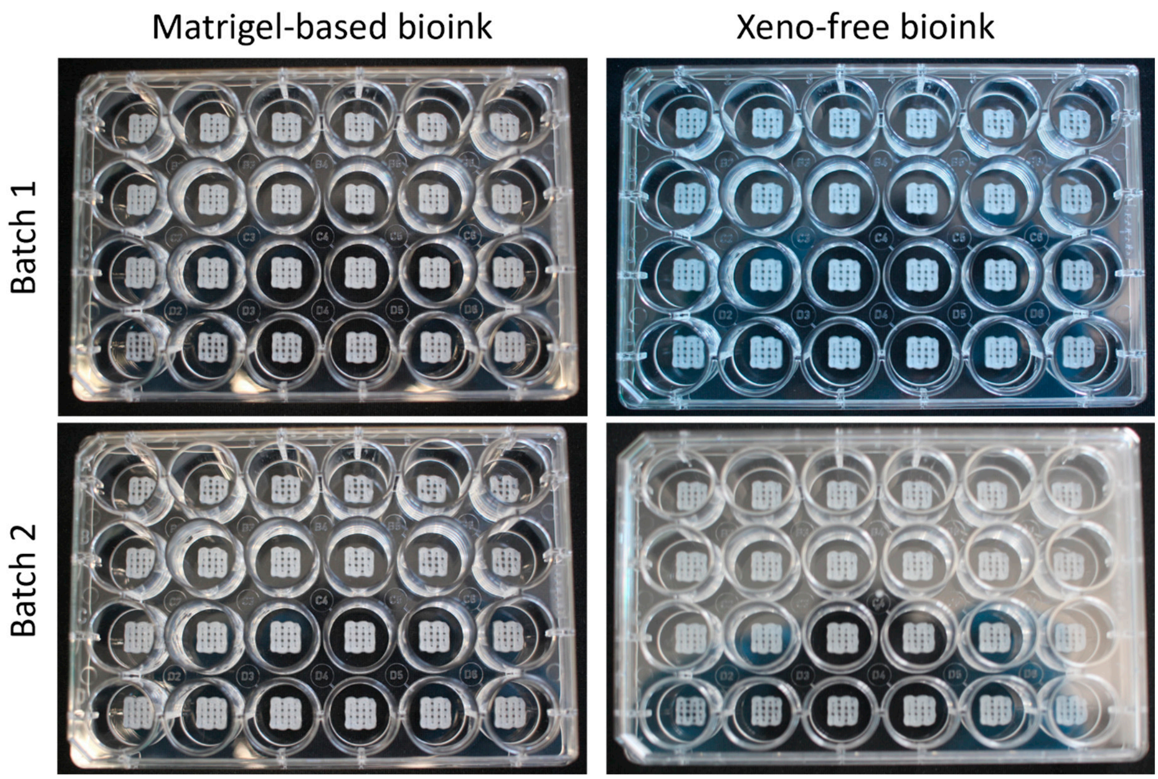

Figure S4. Reproducibility of bioprinting with Matrigel-based bioink and the xeno-free bioink in two different bioprinting batches.

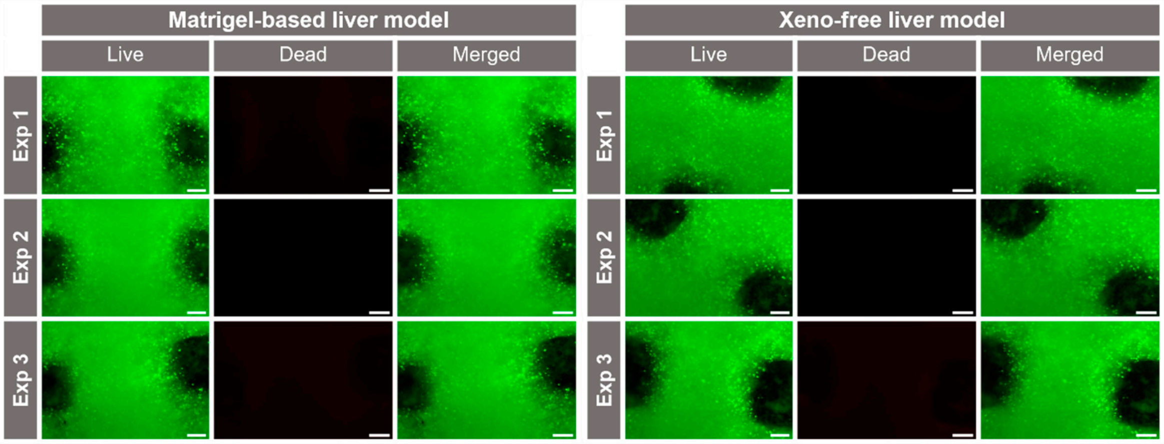

Figure S5. Viability of HuH-7 cells in different batches of 3D printed xeno-free and Matrigel-based liver models. Qualitative viability staining of living and dead HuH-7 cells in bioprinted models stained with calcein-AM (live in green) and ethidium homodimer-1 (dead in red). Scale bar: 200 µm.
